# Long-Term Exposure to Constituents of Fine Particulate Air Pollution and Mortality: Results from the California Teachers Study

**DOI:** 10.1289/ehp.0901181

**Published:** 2009-10-26

**Authors:** Bart Ostro, Michael Lipsett, Peggy Reynolds, Debbie Goldberg, Andrew Hertz, Cynthia Garcia, Katherine D. Henderson, Leslie Bernstein

**Affiliations:** 1 California Environmental Protection Agency, Office of Environmental Health Hazard Assessment, Oakland, California, USA; 2 California Department of Public Health, Richmond, California, USA; 3 Northern California Cancer Center, Berkeley, California, USA; 4 California Air Resources Board, Sacramento, California, USA; 5 City of Hope, Duarte, California, USA

**Keywords:** cardiopulmonary mortality, chronic exposure, cohort study, elemental carbon, fine particles, organic carbon, PM_2.5_, species, sulfates

## Abstract

**Background:**

Several studies have reported associations between long-term exposure to ambient fine particulate matter (PM) and cardiovascular mortality. However, the health impacts of long-term exposure to specific constituents of PM_2.5_ (PM with aerodynamic diameter ≤ 2.5 μm) have not been explored.

**Methods:**

We used data from the California Teachers Study, a prospective cohort of active and former female public school professionals. We developed estimates of long-term exposures to PM_2.5_ and several of its constituents, including elemental carbon, organic carbon (OC), sulfates, nitrates, iron, potassium, silicon, and zinc. Monthly averages of exposure were created using pollution data from June 2002 through July 2007. We included participants whose residential addresses were within 8 and 30 km of a monitor collecting PM_2.5_ constituent data. Hazard ratios (HRs) were estimated for long-term exposure for mortality from all nontraumatic causes, cardiopulmonary disease, ischemic heart disease (IHD), and pulmonary disease.

**Results:**

Approximately 45,000 women with 2,600 deaths lived within 30 km of a monitor. We observed associations of all-cause, cardiopulmonary, and IHD mortality with PM_2.5_ mass and each of its measured constituents, and between pulmonary mortality and several constituents. For example, for cardiopulmonary mortality, HRs for interquartile ranges of PM_2.5_, OC, and sulfates were 1.55 [95% confidence interval (CI), 1.43–1.69], 1.80 (95% CI, 1.68–1.93), and 1.79 (95% CI, 1.58–2.03), respectively. Subsequent analyses indicated that, of the constituents analyzed, OC and sulfates had the strongest associations with all four outcomes.

**Conclusions:**

Long-term exposures to PM_2.5_ and several of its constituents were associated with increased risks of all-cause and cardiopulmonary mortality in this cohort. Constituents derived from combustion of fossil fuel (including diesel), as well as those of crustal origin, were associated with some of the greatest risks. These results provide additional evidence that reduction of ambient PM_2.5_ may provide significant public health benefits.

Several cohort studies have provided evidence linking total and cardiovascular mortality to long-term exposure to fine particulate matter (PM) air pollution [PM ≤ 2.5 μm in diameter (PM_2.5_)] (Abbey et al. 1999; Dockery et al. 1993; Hoek et al. 2002; Laden et al. 2006; Miller et al. 2007; Pope et al. 1995, 2002, 2004). Ambient PM_2.5_ represents a heterogeneous mixture of solid and liquid particles generated by many sources, however, and there is little evidence for which constituents of PM_2.5_ are associated with the greatest risks. Among the available evidence from long-term exposure studies, Pope et al. (1995) and Dockery et al. (1993) both indicated that exposure to fine PM sulfate (SO_4_), likely generated from combusted fossil fuel, was associated with cardiopulmonary mortality.

The U.S. National Research Council has highlighted the importance of investigating characteristics and constituents of PM that contribute to their toxicity (National Research Council 2004). Data regarding differential PM_2.5_ constituent toxicity can have important implications for establishing ambient air quality standards, targeting control strategies, and enhancing the accuracy of health impact assessments. Routine collection of speciated PM_2.5_ data now provides opportunities to examine relationships between long-term exposure to specific PM constituents and morbidity and mortality.

We assessed whether PM_2.5_ constituents were associated with total and cardiopulmonary mortality among participants in the California Teachers Study (CTS), a prospective cohort study of female public school teachers and administrators, initiated in 1995. Within this cohort, the wealth of individual-level data collected allows for control of numerous potential confounders. Active smoking at cohort inception was very low (5%), and indoor occupational exposures among the cohort have generally been low, except to the extent that a small percentage of schools might be located near highways. Thus, residual confounding from smoking or occupational exposures is unlikely. Finally, the large sample size allowed us to restrict the study population to those within close proximity of sites that monitor PM_2.5_ species, thereby reducing potential impacts of pollution measurement error.

## Materials and Methods

### Study population

The CTS is a prospective cohort investigation of 133,479 current and former female public school professionals who completed baseline questionnaires mailed to enrollees in the California State Teachers Retirement System in 1995. Subsequent questionnaires were mailed to CTS participants in 1997 and 2000. Annual follow-up includes updating name and residential information of CTS members and outcome linkages. Participants’ ages varied from 22 to 104 years at enrollment, with a median of 54 years. The cohort is primarily white (86.7%) and born in the United States (93.6%). A full description of the CTS cohort is available elsewhere (Bernstein et al. 2002). All residential addresses from study enrollment forward were geocoded and linked with pollutant data to generate estimates of long-term exposure. Use of data involving human subjects in the CTS was approved by the Committee for the Protection of Human Subjects, California Health and Human Services Agency, and by the institutional review board at each participating institution.

### Health data

CTS records are linked annually to government-maintained mortality and hospitalization files. Data on mortality were obtained from the California Department of Health (2008), the U.S. Social Security Administration death master file (U.S. Department of Commerce, 2008), and the National Death Index (Centers for Disease Control and Prevention 2008). Data on the underlying cause of death from August 2002 through July 2007 were used in our analyses of four mortality categories: all-cause mortality [excluding external causes; all *International Classification of Diseases, 10th Revision* (ICD-10) (World Health Organization 1993), codes except S through Z], cardiopulmonary mortality (ICD-10 codes I00–I99 and J00–J98), mortality due to ischemic heart disease (IHD; ICD-10 codes I20–I25), and mortality due to pulmonary disease (ICD-10 codes J00–J98). Following the practice of the National Center for Health Statistics, coding of cause of death for CTS participants is automated, adhering to World Health Organization rules by using the Automated Classification of Medical Entities system, which eliminates intercoder differences that arise with manual coding. We excluded women who lived outside of California at baseline or whose causes of death were unknown. We restricted our analysis to participants > 30 years of age.

We calculated person-months at risk based on the number of days between 1 August 2002 and *a*) a woman’s date of death, *b*) 31 July 2007, or *c*) the date of first non-California address for women who moved out of state before either *a*) or *b*) occurred.

### Air pollution exposure estimates

PM_2.5_ and species were collected and analyzed by laboratories contracted by the U.S. Environmental Protection Agency (EPA) as part of the Speciation Trends Network (STN) (U.S. EPA 2008). The 24-hr averaged measurements were usually obtained on an every third- or sixth-day basis. Based on prior evidence of associations from time-series studies (Mar et al. 2000; Ostro et al. 2007) and from other epidemiologic or toxicologic studies, we examined PM_2.5_ mass and the following eight constituents: elemental carbon (EC), organic carbon (OC), SO_4_, nitrate (NO_3_), iron (Fe), potassium (K), silicon (Si), and zinc (Zn). Filters were analyzed by EPA staff for EC and OC using the total optical transmittance method; for SO_4_, NO_3_, and K using ion chromatography; and for trace elements using X-ray fluorescence. One monitor in each of the following eight counties collected data on PM_2.5_ and these constituents: Fresno, Kern, Los Angeles, Riverside, Santa Clara, San Diego, Sacramento, and Ventura. All monitors were operating as of 1 June 2002, and data collection for this study ended on 31 July 2007. After that date, changes in collection methods for EC and OC were initiated at several of the sites, rendering comparability with previous measures uncertain.

Each subject was assigned a monthly exposure value based on the monitor nearest her geocoded residential address. Months were included when at least 75% of the potential observations were available. For each individual and each pollutant, the values for all person-months of exposure were summed and then divided by the total months of exposure to create an average measure of overall long-term exposure. Two different exposure assessments were examined. First, we restricted the analyses to subjects whose residences were within 8 km of a monitor. Second, we restricted the analyses to subjects whose residences were within 30 km of a monitor. The smaller distance was selected to minimize potential exposure misclassification while providing a sample size at least as large as that of Harvard Six City (H6C) Study (Dockery et al. 1993), which was among the smallest of the major published air pollution cohort studies. The larger distance was used *a*) to examine the impact on the risk estimates of different distances from the monitor, and therefore different degrees of measurement error, and *b*) to increase the overall study power. Both buffer distances included some participants from counties adjacent to those in which monitors were located.

### Covariates

For covariates, we selected most of the individual-level predictor variables for the regression analysis based on risk factors identified in previous observational studies of the effects of air pollution on cardiovascular and respiratory disease (Dockery et al. 1993; Jerrett et al. 2005; Pope et al. 2002). Sixteen individual-level covariates (a total of 47 terms) were included in the model: marital status; smoking status and pack-years of smoking; second-hand smoke exposure; body mass index (BMI); lifetime physical activity; alcohol consumption; average daily dietary intake of fat, fiber, and calories; menopausal status; hormone replacement therapy use; family history of myocardial infarction or stroke; and use of blood pressure medication or aspirin. Women with BMI < 16 or ≥ 54.9 (< 0.5% of the women) were excluded. In the Cox regression models, described below, the sample was stratified by age (divided into 2-year categories between ages 30 and 79, 3-year categories between ages 80 and 88, and one category for women ≥ 89 years of age) and by race/ethnicity (categorized into three groups: non-Hispanic white, all others, and unknown).

In addition to the individual-level covariates, six ecologic variables were added to the model. These variables, obtained at the census block group level from the 2000 Census (U.S. Census Bureau 2002), were included to consider contextual or neighborhood confounding. The variables, selected based on results from prior analysis (Jerrett et al. 2005), included income (median household income), income inequality (percent living in poverty), education (percent with bachelor’s degree and above), block group population, racial composition (percent black, white, and Hispanic), and unemployment (percent > 16 years of age who were unemployed).

### Statistical methods

The statistical analyses were limited to participants who *a*) were living in California and were at least 30 years of age at baseline, *b*) lived at addresses that were successfully geocoded within either 8 or 30 km of one of the study monitors, *c*) had information available on all continuous variables used in the statistical models, and *d*) did not leave the state for more than four months during the study period.

We used Cox proportional hazards models to estimate hazard ratios (HRs) and 95% confidence intervals (CIs) for each pollutant–outcome combination. Age at the start and end of follow-up (in days) was used to define time on study. We began the exposure period on 1 June 2002—2 months before the start of the cohort follow-up—to allow for analysis of potential impacts from prior exposures. In the analysis, each constituent was examined separately. HRs and the associated 95% CIs were scaled to the interquartile range (IQR), based on the average distributions for each pollutant for all women. Three different models were run, all adjusted for age and race. The first model included only a pollutant variable, each entered in a separate regression that was otherwise unadjusted. A second model included the full set of individual-level risk factors summarized above. The final model also included all of the contextual variables to test for the influence of local geographic impacts. Because there was little difference in the risk estimates for the unadjusted models versus those adjusted for individual covariates and those adjusted for both individual covariates and contextual risk factors, we present only the results for the fully adjusted models. However, the results of all three models using OC as the pollutant are presented in the Supplemental Material [see Table S-1 (doi:10.1289/ehp.0901181.S1 via http://dx.doi.org)]. As indicated above, we examined the risks for participants within either 8 or 30 km of the PM_2.5_ species monitors. Also, as a sensitivity analysis, we conducted a forward-selection regression procedure of the PM_2.5_ constituents to determine their relative importance. Because of the likelihood of multicollinearity, we constrained the selection to include only positive coefficients with *p* < 0.05. Several other sensitivity analyses were conducted using cardiopulmonary mortality as the end point. First, we examined several two-pollutant models focusing on those pollutants that appeared to be most strongly associated with mortality. Second, we examined the implications of using a full year of exposure data (rather than only 2 months) before the follow-up period. Thus, in this analysis, exposures began in June 2002 but cohort follow-up began in June 2003 (rather than August 2002 as in the base case). Both the exposure and cohort follow-up continued through July 2007. Finally, we examined the effects of using an exposure estimate consisting of only a single annual average for each pollutant, similar to some previous studies (Miller et al. 2007). In this case, the exposures consisted of a given pollutant’s annual average for the period June 2002 through May 2003 based on the monitor at the participants’ residence at the start of the follow-up period (June 2003). Proportional hazards analyses were conducted using SAS software (version 9.1; SAS Institute Inc., Cary, NC).

## Results

A total of 9,208 participants resided within 8 km of a monitor, and 52,226 within 30 km. About 5% of each group were < 30 years of age, 5% moved or died before August 2002, 3% were missing individual-level risk factors, and 1% were missing contextual risk factors. This left totals of 7,888 and 44,847 participants within 8 and 30 km of a monitor, respectively. Table 1 summarizes the pollutant variables used in the analyses and provides the mean, minimum, maximum, and IQR for each. Measures are based on individual-level estimates of exposure and do not represent the average readings for any specific monitor or set of monitors. The long-term mean PM_2.5_ concentrations for the participants within 8 km and 30 km were 17.0 and 17.5 μg/m^3^, respectively. For each buffer designation, OC constituted about one-third and NO_3_ about one-fourth of the total PM_2.5_ mass.

Table 2 summarizes some of the individual baseline characteristics of the CTS participants eligible for the analysis and living within the 8- and 30-km buffers, as well as their residential locations, by county. Individual characteristics (e.g., age, BMI, smoking status, marital status, menopausal status, hormone therapy use, and family history of heart disease) were similar for the two buffer configurations. Thirteen percent of all respondents within the 8-km buffers and 36% within the 30-km buffers of the species monitors resided in Los Angeles County.

Table 3 summarizes the correlations of the individual-level exposures to the constituents of PM_2.5_ based on the 8- and 30-km buffers around the species monitors. We observed relatively high correlations (*r* > 0.8) between PM_2.5_ and EC, NO_3_, and Zn. Among the constituents, we observed high correlations (*r* > 0.8) between EC and NO_3_, Fe, and Zn; between OC and K; and between Zn and NO_3_ and Fe.

The results of the regression analyses are presented in Tables 4 and 5 for the 8-km and 30-km buffers, respectively. For the 8-km buffer, 58% of all deaths were from cardiopulmonary disease versus 54% for the 30-km buffer. In addition, 6.8% of all women in the 8-km buffer and 5.8% of women in 30-km buffer died during the 5-year follow-up period. In the regression analysis, we entered each constituent separately into the model. Using a buffer of 8 km around each species monitor, we observed associations with all-cause, cardiopulmonary, and IHD mortality for PM_2.5_ mass and all of the constituents. For pulmonary mortality, we observed significant associations for OC, SO_4_, and Si but not PM_2.5_. We generally observed modestly higher HRs for cardiopulmonary versus all-cause mortality for PM_2.5_ mass and several constituents, whereas those for IHD were markedly greater. In addition, in micrograms per cubic meter, all of the constituents had greater HRs than did PM_2.5_ mass (data not shown).

Using the 30-km buffer around each PM_2.5_ species monitor (Table 5), we again observed associations between most of the constituents and all-cause, cardiopulmonary, and IHD mortality. EC was not associated with all-cause or cardiopulmonary mortality but was associated with IHD mortality. For pulmonary mortality, we observed associations for PM_2.5_ mass, OC, SO_4_, NO_3_, and Si. With few exceptions, the HRs were similar to or slightly less than those observed with the 8-km buffer.

Using the 8-km buffer, we conducted a forward-selection regression procedure to determine the relative importance of the constituents. For all four outcomes, only two constituents, OC followed by SO_4_, successfully entered the model. Forcing additional constituents into the model generated clear signs of multicollinearity, including widely varying (and high) HRs and negative β-coefficients. When PM_2.5_ was also included as a candidate variable for inclusion, OC and SO_4_ were still selected first in the model.

Table 6 summarizes the results of two-pollutant models for cardiopulmonary mortality using the 8-km buffer and including each of the other constituents in the model specification with either OC or SO_4_. In the models with OC plus another constituent, OC remained associated with cardiopulmonary mortality, with a fairly consistent HR. Likewise, in the models with SO_4_ plus another constituent, SO_4_ was consistently associated with cardiopulmonary mortality with an HR similar to models in which SO_4_ was the only pollutant.

**Figure 1:**
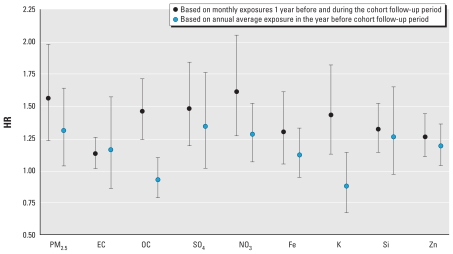
Association of cardiopulmonary mortality with PM_2.5_ and its constituents using alternative exposure metrics: HR and 95% CI for 8-km buffer and cohort follow-up from June 2003 through July 2007.

We conducted an additional sensitivity analysis using exposures starting a full year before the beginning of the follow-up period. The exposures were calculated monthly from June 2002 through July 2007, whereas the cohort follow-up period was June 2003 through July 2007. The results were essentially unchanged from the basic model when the follow-up period began in August 2002, as summarized in Table 4 [see Supplemental Material, Table S-2 (doi:10.1289/ehp.0901181.S1)]. The two exceptions were reductions in HRs for OC (HR = 1.46; 95% CI, 1.24–1.71) and K (HR = 1.43; 95% CI, 1.13–1.82), with the latter becoming more consistent with the other estimates. In our final sensitivity analysis, we considered a single annual average (June 2002 through May 2003) as our exposure metric using the reported residence of each study participant for June 2003. We used a cohort follow-up period similar to the previous analysis (i.e., June 2003 through July 2007). As summarized in Figure 1, the HRs for cardiopulmonary mortality were reduced for each of the constituents (except for EC, for which the HR was similar), and we observed significant associations only for PM_2.5_, SO_4_, NO_3_, and Zn.

## Discussion

In this study we found strong and consistent associations between long-term exposure to PM_2.5_ mass, as well as several of its constituents, and all-cause, cardiopulmonary, IHD, and pulmonary mortality. Specifically, in single-pollutant models using the 8-km buffer, PM_2.5_ mass and all of the constituents were associated with all-cause, cardiopulmonary, and IHD mortality. For pulmonary mortality, we observed associations for OC, SO_4_, NO_3_, and Si but not for PM_2.5_ mass. When a buffer size of 30 km was used, we observed generally similar results except that EC was no longer associated with all-cause or cardiopulmonary mortality and PM_2.5_ was associated with pulmonary mortality. Forward-selection regression analysis indicated that long-term exposures to OC and SO_4_, in particular, had stronger associations with all four outcomes than did the other constituents or PM_2.5_ mass. Subsequent analysis of cardiopulmonary mortality using two-pollutant models that included either OC or SO_4_ plus one other constituent provided additional support for the importance of these two pollutants, because their HRs were robust to inclusion of other pollutants in the model.

We focused particular attention on attempting to improve the exposure assessment by using information on monthly residential history of the cohort and by limiting the sample to 8- or 30-km buffers around each monitor. Comparing results between the 8- and 30-km buffers, we observed lower HRs and a small reduction in the number of constituents associated with the mortality outcomes, which would be expected from increased exposure measurement error. We also conducted several sensitivity analyses of our exposure metric. The results differed little when we started the exposure period 2 months or 12 months before the initiation of cohort follow-up. However, we observed significant differences in the results when participants were all assigned a single annual average exposure based on their residential location in the first month of the follow-up period. It is not clear whether this result would hold for other data sets, but it merits further examination.

To our knowledge, only a few previous studies of long-term exposure have examined the effects of any constituents of PM_2.5_. In analyses of the cohorts from the ACS and the H6C Study, Pope et al. (1995, 2002) and Dockery et al. (1993) reported associations between SO_4_ and both all-cause and cardiopulmonary mortality. In addition, a cross-sectional study of mortality in the United States reported associations between metropolitan area-wide mortality rates and SO_4_ (Özkaynak and Thurston 1987). Using both the 8-km and 30-km buffer, we also found associations between SO_4_ and all four outcomes. In addition, in our forward stepwise selection regressions, SO_4_ was selected to be included in the model for all four outcomes. Thus, long-term exposure to SO_4_ is consistently associated with mortality even at the relatively low concentrations observed in this study (mean of 2.5 μg/m^3^ using the 30-km buffer) relative to those reported by Pope et al. (1995) and Dockery et al. (1993) (~ 11 and 8 μg/m^3^, respectively). Besides low-sulfur motor vehicle fuels and diesel-powered ships, there are relatively few sources of sulfur emissions in California. Therefore, SO_4_ makes up a much smaller proportion of PM_2.5_ in California than in the eastern or midwestern United States (Bell et al. 2007).

Regarding other constituents of PM_2.5_, a Dutch cohort study included an examination of relationships between mortality and long-term exposure to black smoke, a primary pollutant measured by reflectance on a Teflon filter, which has been shown to be highly correlated with EC (Beelen et al. 2008; Cyrys et al. 2003). In that study, associations were reported between black smoke and all-cause mortality and less so with cardiovascular mortality. In the CTS cohort using the 8-km buffer, we also observed associations for EC. However, we observed no association using the 30-km buffer, which may have been due in part to significant exposure misclassification. In general, researchers have observed exponential declines in EC concentrations with downwind distance from roadways. For instance, in Los Angeles EC drops to background levels within 100–150 m downwind from busy roads (Zhu et al. 2002). Wind speed and direction can significantly affect EC concentrations; PM_2.5_ concentrations 150 m from the highway can be reduced by 50% depending on the wind direction (Hitchins et al. 2000).

We observed associations for OC with all four outcomes that we examined using both buffers. Moreover, using a forward-selection procedure, OC was the first pollutant selected for model inclusion for all-cause, cardiopulmonary, and IHD mortality (even when PM_2.5_ was included as a candidate pollutant). OC is both directly emitted and, after transformation through atmospheric chemical reactions, a secondary product of fuel combustion. Key sources in California include gasoline and diesel vehicles, residential wood combustion, agricultural and prescribed burning, and industrial combustion of fossil fuels. Diesel PM_2.5_ emissions consist of both OC and EC fractions, along with trace amounts of inorganic compounds (Abu-Allaban et al. 2004). The OC fraction of diesel exhaust contains heavy hydrocarbons, such as lubricating oils and non- and semivolatile polycyclic aromatic hydrocarbons. Although no previous epidemiologic studies of long-term exposure have included OC, there is evidence of serious health effects related to short-term exposures. For example, in studies of daily exposures in six California counties and in Phoenix, Arizona (Mar et al. 2000; Ostro et al. 2007), associations were reported between OC and cardiovascular mortality. In another study of short-term exposures, Metzger et al. (2004) reported an association between OC and emergency department visits for cardiovascular disease in Atlanta, Georgia. Possible pathophysiologic mechanisms linking OC with mortality, especially from IHD, may include oxidative stress and effects on blood pressure (Brook et al. 2004).

In single-pollutant models, we also observed associations of all-cause, cardiopulmonary, and IHD mortality with NO_3_, K, Fe, and Zn. K is often used as a marker of biomass combustion and residential wood burning, which tend to be a local and neighborhood problem in several parts of California (Lipsett et al. 1997). Because of high correlations with other pollutants in our study, however, it is difficult to determine whether long-term exposures to these constituents have independent effects on health. Previous toxicologic and epidemiologic studies, however, provide support for significant cardiovascular effects from exposure to all four of these constituents (Brook et al. 2004; Fairley 2003; Hoek 2003; Naeher et al. 2007; Ostro et al. 2007).

We observed strong associations between Si and several mortality categories, especially pulmonary mortality. Si is a crustal element that is a large component of soil and resuspended road dust. As such, it may be enriched by and serve as a surrogate for many toxic constituents found in road dust, including combustion-based material, brake dust, tire debris, and semivolatile compounds (Rogge et al. 1993). It may also serve as a general marker for proximity to traffic. Several studies of long-term exposure to traffic or traffic-based pollutants have reported associations with cardiopulmonary mortality (Beelen et al. 2008; Filleul et al. 2005; Jerrett et al. 2005) and with the development of cardiovascular disease (Hoffmann et al. 2006). Finally, a few studies have reported cardiovascular effects from direct exposure to Si. For example, Si has been found to be associated with *a*) ST-segment elevation, a marker for myocardial ischemia, in dogs; *b*) vasoconstriction of pulmonary arteries in rats; *c*) heart rate variability in humans; and *d*) cardiovascular mortality in time-series studies in Arizona and California (Batalha et al. 2002; Cavallari et al. 2008; Lipsett et al. 2006; Mar et al. 2000; Ostro et al. 2007; Wellenius et al. 2003). In contrast, in a study of six eastern and midwestern cities in the United States, the authors did not observe an association between mortality and daily exposure to Si (Laden et al. 2000). Thus, whereas it is likely that Si serves as a proxy either for the toxic constituents found in road dust or for exposures to traffic-related pollutants, the possibility of direct cardiotoxicity, although unlikely at the low concentrations observed in this investigation, cannot be ruled out.

Our findings of an association between long-term exposure to PM_2.5_ and all-cause and cardiovascular mortality in women are consistent with those reported in previous studies (Chen et al. 2005; Dockery et al. 1993; Eftim et al. 2008; Krewski et al. 2000; Miller et al. 2007; Pope et al. 1995, 2002, 2004). Several studies reported similar relative risks for females and males. However, our estimated HRs for the all-female teacher cohort were generally higher than results present for most other female cohorts, with the exception of the recent study of the Women’s Health Initiative (WHI) observational study (Miller et al. 2007). For cardiopulmonary mortality in females associated with a 10 μg/m^3^ change in PM_2.5_, HRs of 1.16 (95% CI, 1.08–1.27), 1.20 (95% CI, 1.09–1.32), and 1.76 (95% CI, 1.25–2.47) were reported for cohorts from the ACS, H6C Study, and WHI (for cardiovascular mortality), respectively (Dockery et al. 1993; Krewski et al. 2000; Miller et al. 2007; Pope et al. 1995). In contrast, extrapolating from the 30-km buffer results in the present study, a 10-μg/m^3^ change in PM_2.5_ is associated with an HR of 2.05 (95% CI, 1.80–2.36). There are several possible explanations for the higher estimate in this study. First, it may be attributable to efforts to improve the exposure assessment, because our study incorporated monthly residential history and small buffers around each pollution monitor. Second, the overall exposures may be greater because of the mild climate in California, where people are likely to have their windows open and spend more time outdoors. Finally, the greater relative risk estimates may be simply attributable to stochastic variability.

We observed particularly high relative risks from exposure to PM_2.5_ mass and several of its constituents for mortality from IHD. For example, using the 8-km buffer, the HRs associated with the IQRs for PM_2.5_, OC, and SO_4_ were 2.10, 2.02, and 1.82, respectively. Several other studies have reported associations for this outcome, as well (Jerrett et al. 2005; Miller et al. 2007; Pope et al. 2002).

This study is subject to several potential limitations. First, the study cohort may not be representative of the general population of California women or the adult female population of the United States. Likewise, the mix of pollutants in California is different from that observed in the U.S. Midwest and East Coast, because traffic is the dominant source of PM in California and there are few emissions from major industrial facilities such as coal-fired power plants. Third, we were limited to contemporaneous exposures for the PM_2.5_ species, which could bias the ultimate effect estimates if historical exposures are more important. Fourth, we based exposure assessment only linkage of nearest fixed-site monitors with residence; exposures derived from sources such as motor vehicle exhaust while commuting or indoor environments at homes and schools could not be accounted for in this data set. Fifth, exposure misclassification was likely lower within the smaller buffer radius, but this came at the cost of reduced statistical power. Few IHD and pulmonary events occurred among women within the 8-km buffer, which produced somewhat imprecise estimates. Finally, some caution is warranted in attributing a causal relationship to any single constituent because its effect estimate may be attributable to its intrinsic toxicity or its correlation with other, more toxic substances. In addition, the constituents may have differential measurement error due to differences in spatial variability and in indoor/outdoor penetration. Additional research using more years for this cohort, when available, or from other studies will be necessary to confirm the importance of the constituents associated with higher HRs in this investigation.

In summary, we analyzed a relatively homogeneous cohort of female teachers and administrators, coupled with spatial restrictions to reduce the effects of exposure misclassification. The results provide evidence for effects from long-term exposures to PM_2.5_ and several of its constituents on mortality. The constituents that appear to generate most of the risk are derived from combustion of fossil fuel (including diesel) and biomass, as well as from PM of crustal origin. Reduction of ambient PM_2.5_, particularly from fuel combustion, may provide significant public health benefits.

## Figures and Tables

**Table 1:** Descriptive statistics of individual-level pollutant exposures (μg/m^3^) among participants in the CTS cohort, 1 June 2002 through 31 July 2007 (24-hr averages).

	Participants within 8-km buffer (*n* = 7,888)			Participants within 30-km buffer (*n* = 44,847)		
Pollutant	Mean	IQR	Range	Mean	IQR	Range
PM_2.5_	17.0	6.1	7.6–34.7	17.5	6.1	6.8–38.7
EC	1.0	0.16	0.28–1.8	1.1	0.65	0.20–2.4
OC	6.1	1.0	3.1–12.1	5.9	0.83	2.1–10.1
SO_4_	2.0	1.3	0.6–7.4	2.5	2.2	0.62–7.4
NO_3_	4.5	3.6	0.7–14.9	4.9	3.2	0.7–16.2
Fe	0.12	0.06	0.05–0.34	0.14	0.13	0.04–0.36
K	0.11	0.05	0.04–0.35	0.11	0.07	0.02–0.35
Si	0.14	0.05	0.04–0.40	0.13	0.03	0.03–0.49
Zn	0.01	0.01	0.00–0.03	0.01	0.01	0.00–0.04

**Table 2:** Baseline characteristics of the CTS participants whose residences were within 8-km and 30-km buffers around PM_2.5_ species monitors, August 2002–July 2007.

Individual characteristics	Species 8-km buffer (*n* = 7,888)	Species 30-km buffer (*n* = 44,847)
Age at intake [years (mean ± SD)]	54.3 ± 13.4	53.4 ± 13.0
Race (% white)	85.9	83.4
BMI [mean (kg/m^2^)]	25.2	25.1
Average dietary fat intake (g/day)	56.2	55.8
Smoking status (%)		
Never smoker	68.5	68.3
Former smoker	26.1	26.9
Current smoker	5.4	4.9
Married (%)	42.7	45.1
Menopausal status (%)		
Premenopausal	36.2	38.3
Peri/postmenopausal and no hormone therapy use	13.6	13.0
Peri/postmenopausal and current/past hormone therapy use	38.6	37.3
Unknown menopausal status/hormone therapy use	11.6	11.4
Family history of heart disease (%)	33.8	34.8
Mean daily dietary calories (kcal)	1,584	1,579
Average no. of pack-years among former and current smokers	15.6	14.6
Adult second-hand smoke exposure (%)	51.1	49.1
Nondrinker (%)	34.7	34.2
Participant locations (proportion within each county)		
Alameda County	0.3	1.7
Fresno County	16.0	5.8
Kern County	12.0	3.3
Los Angeles County	13.3	36.4
Riverside County	6.6	4.4
Sacramento County	18.3	8.4
San Bernardino County	0.6	7.0
San Diego County	14.1	13.6
Santa Clara County	12.7	10.4
Ventura County	3.0	3.0
Other	3.1	6.0

**Table 3:** Correlations among PM_2.5_ mass and constituents based on individual-level exposure assessment for the participants within 8-km (top line in each row) and 30-km (bottom line in each row) buffers.

	PM_2.5_	EC	OC	SO_4_	NO_3_	Fe	K	Si	Zn
PM_2.5_	1.00	0.84	0.60	0.59	0.89	0.71	0.67	0.78	0.84
		0.82	0.64	0.72	0.90	0.76	0.66	0.80	0.91

EC		1.00	0.51	0.53	0.82	0.91	0.64	0.58	0.92
			0.67	0.73	0.74	0.96	0.80	0.58	0.90

OC			1.00	−0.10	0.44	0.31	0.84	0.73	0.55
				0.29	0.48	0.55	0.87	0.71	0.61

SO_4_				1.00	0.73	0.60	0.10	0.27	0.54
					0.79	0.79	0.47	0.45	0.67

NO_3_					1.00	0.79	0.50	0.74	0.83
						0.76	0.48	0.79	0.85

Fe						1.00	0.50	0.51	0.85
							0.70	0.53	0.87

K							1.00	0.71	0.60
								0.61	0.65

Si								1.00	0.60
									0.70

Zn									1.00

**Table 4:** Association between mortality outcomes and PM_2.5_ and its constituents using 8-km buffer [HRs (95% CIs) for the IQR of each pollutant].

Pollutant	IQR (μg/m^3^)	All cause (*n* = 540)	Cardiopulmonary (*n* = 312)	IHD (*n* = 110)	Pulmonary (*n* = 81)
PM_2.5_	6.1	1.49 (1.28–1.74)	1.58 (1.29–1.93)	2.10 (1.49–2.97)	1.39 (0.91–2.11)
EC	0.16	1.10 (1.03–1.19)	1.11 (1.00–1.22)	1.26 (1.07–1.48)	0.94 (0.75–1.16)
OC	1.0	1.70 (1.53–1.87)	1.64 (1.44–1.87)	2.02 (1.62–2.51)	1.55 (1.18–2.02)
SO_4_	1.3	1.49 (1.30–1.71)	1.54 (1.28–1.85)	1.82 (1.33–2.50)	1.61 (1.13–2.31)
NO_3_	3.6	1.40 (1.20–1.65)	1.53 (1.24–1.88)	1.86 (1.31–2.65)	1.39 (0.90–2.13)
Fe	0.06	1.23 (1.06–1.42)	1.26 (1.04–1.52)	1.61 (1.17–2.20)	1.02 (0.68–1.53)
K	0.05	1.90 (1.63–2.21)	1.72 (1.40–2.11)	2.59 (1.79–3.73)	1.22 (0.82–1.82)
Si	0.05	1.36 (1.25–1.49)	1.44 (1.28–1.62)	1.57 (1.27–1.94)	1.43 (1.14–1.81)
Zn	0.01	1.16 (1.06–1.27)	1.18 (1.05–1.33)	1.29 (1.06–1.56)	1.09 (0.84–1.41)

All models are adjusted for smoking status, total pack-years, BMI, marital status, alcohol consumption, second-hand smoke exposure at home, dietary fat, dietary fiber, dietary calories, physical activity, menopausal status, hormone replacement therapy use, family history of myocardial infarction or stroke, blood pressure medication and aspirin use, and contextual variables (income, income inequality, education, population size, racial composition, unemployment).

**Table 5:** Association between mortality outcomes and PM_2.5_ and its constituents using 30-km buffer [HRs (95% CIs) for the IQR of each pollutant].

Pollutant	IQR (μg/m^3^)	All cause (*n* = 2,590)	Cardiopulmonary (*n* = 1,397)	IHD (*n* = 474)	Pulmonary (*n* = 366)
PM_2.5_	6.1	1.45 (1.36–1.55)	1.55 (1.43–1.69)	1.91 (1.65–2.21)	1.43 (1.21–1.69)
EC	0.65	1.04 (0.95–1.14)	1.07 (0.95–1.21)	1.41 (1.14–1.74)	0.91 (0.71–1.17)
OC	0.83	1.73 (1.64–1.82)	1.80 (1.68–1.93)	2.03 (1.79–2.29)	1.73 (1.51–1.97)
SO_4_	2.2	1.67 (1.52–1.83)	1.79 (1.58–2.03)	2.39 (1.93–2.97)	1.59 (1.24–2.03)
NO_3_	3.2	1.32 (1.24–1.39)	1.40 (1.29–1.51)	1.66 (1.46–1.90)	1.31 (1.13–1.52)
Fe	0.13	1.22 (1.12–1.34)	1.25 (1.10–1.42)	1.66 (1.34–2.05)	1.05 (0.82–1.35)
K	0.07	1.43 (1.31–1.55)	1.50 (1.34–1.68)	2.06 (1.70–2.49)	1.24 (0.99–1.55)
Si	0.03	1.36 (1.32–1.40)	1.39 (1.33–1.45)	1.47 (1.37–1.59)	1.35 (1.23–1.47)
Zn	0.01	1.15 (1.07–1.25)	1.21 (1.09–1.35)	1.52 (1.27–1.82)	1.06 (0.86–1.31)

All models are adjusted for smoking status, total pack-years, BMI, marital status, alcohol consumption, second-hand smoke exposure at home, dietary fat, dietary fiber, dietary calories, physical activity, menopausal status, hormone replacement therapy use, family history of myocardial infarction or stroke, blood pressure medication and aspirin use, and contextual variables (income, income inequality, education, population size, racial composition, unemployment).

**Table 6:** Associations with cardiopulmonary mortality using two-pollutant models and the 8-km buffer.

Model	HR (95% CI)
OC	1.64 (1.44–1.87)

OC	1.67 (1.45–1.92)
EC	0.97 (0.87–1.08)

OC	1.81 (1.59–2.06)
SO_4_	1.90 (1.57–2.29)

OC	1.59 (1.38–1.84)
NO_3_	1.13 (0.88–1.44)

OC	1.63 (1.43–1.86)
Fe	1.19 (0.98–1.44)

OC	1.69 (1.37–2.08)
K	0.94 (0.67–1.31)

OC	1.55 (1.34–1.78)
Si	1.29 (1.14–1.46)

OC	1.62 (1.41–1.86)
Zn	1.04 (0.91–1.19)

SO_4_	1.54 (1.28–1.85)

SO_4_	1.61 (1.29–2.00)
EC	0.96 (0.85–1.08)

SO_4_	1.90 (1.57–2.29)
OC	1.81 (1.59–2.06)

SO_4_	1.39 (1.13–1.72)
NO_3_	1.27 (1.00–1.61)

SO_4_	1.45 (1.14–1.83)
Fe	0.90 (0.70–1.15)

SO_4_	1.53 (1.27–1.84)
K	1.68 (1.37–2.04)

SO_4_	1.48 (1.22–1.79)
Si	1.41 (1.26–1.59)

SO_4_	1.51 (1.21–1.87)
Zn	1.02 (0.89–1.18)
